# Surprise! Why Insightful Solution Is Pleasurable

**DOI:** 10.3390/jintelligence10040098

**Published:** 2022-11-07

**Authors:** Anna Savinova, Sergei Korovkin

**Affiliations:** 1 Laboratory for Cognitive Research, Yaroslavl State University, pr-d Matrosova, 9, 204, 150057 Yaroslavl, Russia; 2 Department of Psychology, Yaroslavl State University, pr-d Matrosova, 9, 204, 150057 Yaroslavl, Russia

**Keywords:** insight, insight problem solving, Aha! experience, expectation, prediction error

## Abstract

Insight problems—as a type of ill-defined problems—are often solved without an articulate plan, and finding their solution is accompanied by the Aha! experience (positive feeling from suddenly finding a solution). However, the solution of such problems can also be guided, for example, by expectations in terms of criteria for achieving the goal. We hypothesize that adjusting the expectation accuracy based on the reward prediction error (discrepancy between the reward and its prediction) affects the strength of affective components of the Aha! experience (pleasure and surprise), allowing to learn how to solve similar problems. We manipulated expectation accuracy by varying the similarity in problem solution principle and structure in a short learning set. Each set was followed by a critical problem where both the structure and solution principle were changed (except for control set). Subjective feelings, solution time, and expectation were measured after each problem. The results revealed that problems with similarities become more expected at the end of the set and their solution time is decreased. However, the critical problem featured a rapid increase in pleasure and surprise and decrease in expectedness only in the condition where both the solution principle and structure were expected, suggesting that problem structure is a key feature determining expectedness in insight problem solving. The Aha! experience is not an epiphenomenon; it plays a role in learning of problem solving through adjusting expectations.

## 1. Introduction

Can people enjoy intellectual failure? Such pleasure seemingly makes no sense; however, insight problems, magic tricks, and jokes are often experienced that way. When faced with, at first glance, insurmountable difficulties, people end up experiencing pleasure from solving the problem. They even attempt to replicate this feeling in the future. Insight, humor, puzzles, and charades are somewhat of a social game: while some people create them, others actively explore and solve them. These games are rooted in playing with others’ expectations (Airenti 2016). Delight and surprise in magic tricks are usually caused by an inexplicable and unexpected event that happened in front of the audience (Danek et al. 2015), witticisms in verbal jokes are built on an unexpected turn in the statement (Attardo and Raskin 1991), and unexpected music elements attract listeners (Huron 2006). These are all examples that can be attributed to a wide class of ‘pleasures of the mind’ phenomena that arise due to changes in emotions as a result of a violation of expectations and the need to adjust one’s experience (Kubovy 1999; Bianchi et al. 2022; Canestrari et al. 2018). In these examples, positive emotions are elicited by something different from the usual context and at the same time still located within the zone of expectations.

One such phenomena in which pleasure and surprise are manifested due to incorrect expectations is insight. An insight solution is often described as follows: a solver is faced with the problem, builds an incorrect representation that does not allow them to find the solution, and ends up in an impasse. The solver then needs to change the representation by restructuring the problem to overcome the impasse (Ohlsson 1992, 2011). This description contains both cognitive (the impasse, the representational change) and affective components (the emotionally pleasant Aha! experience). The subjective feeling accompanying the restructuring, which is usually referred to as an Aha! experience, is experienced suddenly and unexpectedly and brings pleasure to the solver (Metcalfe and Wiebe 1987). The Aha! experience also accompanies the correct solution (Danek et al. 2014; Salvi et al. 2016; Webb et al. 2016, 2018; Laukkonen et al. 2021, 2022). Interestingly, this relationship is dynamic: the greater the strength of the Aha! experience, the more likely the solution will be correct (Danek and Wiley 2017; Danek et al. 2020; Threadgold et al. 2018; Webb et al. 2016; Laukkonen et al. 2021). At the same time, the Aha! experience not only accompanies correct ideas, but can be overgeneralized and related to facts that were in the solver’s mind at that moment. For example, it reinforces false beliefs and delusions (Laukkonen et al. 2020, 2022). In essence, the Aha! experience rewards self-dependent, endogenous solutions because the solver’s time and effort were spent on it (Kizilirmak et al. 2021). It might be the case that the Aha! experience is not just a reinforcement of the self-dependent solution, but a reaction to the expectation violation.

A possible function of the Aha! experience could be reinforcement of the useful experience gained as a result of the solution (Auble et al. 1979; Danek et al. 2013; Danek and Wiley 2020). Ohlsson (2011) theoretically considers insight as non-monotonic learning. Unlike classical monotonic learning, in which monotonously repetitive events and patterns are learned, non-monotonic learning occurs as an abrupt restructuring of experience as a result of a collision with changed environment. Thus, insight is necessary to acquire and consolidate new useful experience into memory, or rather to adjust past experience in accordance with new conditions. In support of this idea, Danek and colleagues demonstrated that the Aha! experience makes it easier to recall the solutions of magic tricks (Danek et al. 2013). This effect could be caused both by the cognitive effect of a solution transfer after restructuring, and an affective reaction accompanying the solution. Later, Danek and Wiley (2020) showed that the effect of facilitating the solution recall is associated with the joint influence of these factors.

In this paper, we hypothesize that the Aha! experience is not only reinforcement of the useful experience, but it also changes depending on the solution familiarity. The reward prediction error approach could better explain this connection. Recently, Dubey et al. (2022) proposed a theoretical framework linking the intensity of the Aha! experience with the surprise of a solution based on the idea of learning by reward prediction errors. Within this framework, learning occurs as a result of the discrepancy between the reward and its prediction (reward prediction error), so that an unpredicted reward reinforces the behavior that preceded it, a fully predicted reward does not cause a response, and the absence of a predicted reward causes a negative reinforcement (Schultz 2016). Such reinforcement may be characteristic in general for any prediction errors (Clark 2013; Friston 2010). If one’s predictions turn out to be accurate, this is a signal that the knowledge adequately corresponds to the problems being solved. In case of a prediction error, deviation from expectations, there is a need to adjust experience and knowledge. Moreover, small deviations from predictions can be accompanied by a positive effect, which reinforces learning more strongly. Chetverikov and Kristjánsson (2016) suggested that affect serves as feedback for predictions, evaluates predictions’ accuracy, and regulates predictions by promoting the most successful ones. From this point of view, the Aha! experience is an affective reaction to the discovery of a solution that differs from the expected one. This affective reaction signals the need to learn new knowledge and correct expectations.

Experiences of surprise in insightful solutions may indicate that the solvers are guided by some initial expectations or predictions about the solution. Some previously obtained data can be interpreted from the point of view of the reward prediction error learning approach. Ammalainen and Moroshkina (2021) showed that participants rated their solutions as more insightful when hints were not provided compared to the conditions where hints were present. Arguably, hints make the solution more expected but, as a result, less insightful. Bilalić et al. (2019) demonstrated that past problem-solving experience also decreases the strength of the Aha! experience (was measured by multiple subjective scales). Chess players solve a chess-related insight problem (the checkerboard problem) faster than non-chess players. Chess players reported less pleasure, surprise, and sudden solutions in checkerboard problems, although they felt more certainty in the solution than among non-chess players. However, insight problems in which chess knowledge was not relevant to building accurate problem representation (the eight-coins problem) were not different in terms of the solution effectiveness and the strength of the Aha! experience between the chess players and non-chess players. According to the error prediction approach, chess players make more accurate predictions about the solution of chess-related problems, hence their solutions are more obvious and expected.

A prediction error in solving a problem can be associated with at least two types of expectations: metacognitive predictions of the solver’s success and cognitive predictions concerning progress within the problem space. The solution may be unexpected for the solvers in the sense that they did not expect to solve the problem, or it may contain unexpected content.

Metacognitive account was developed by Dubey et al. (2022) by linking the intensity of the Aha! experience with metacognitive predictions of the ability to solve the problem and expected duration of the problem solution. They proposed a computational metacognitive model of the Aha! experience based on the metacognitive prediction error. Within the proposed model, the solvers monitor their success in problem solving in order to predict the time it will take them to complete the task. The greater the positive prediction error of success, the more intense the Aha! experience.

The metacognitive approach predicts quite reasonably that the Aha! experience can be found not only in solving classical insight problems, but also in various other situations: feeling of the tip-of-the-tongue, magic tricks, categorization in difficult conditions, etc. However, such a model assumes only the adjustment of metacognitive expectations about solvers’ abilities but does not clarify the question of how this can contribute to learning to solve problems.

In this paper, we propose an approach in which the reward prediction errors are considered as criteria for achieving the goal during problem solving. We are addressing the question of what role the Aha! experience can play within the process of problem solving. The problem-solving process can be considered as a progress from the initial state to the goal state (Newell and Simon 1972). The progress is achieved by reducing the differences between the current and the anticipated goal state. This idea fits well-defined problems in which the conditions and goals are clearly defined. Chronicle, MacGregor, and Ormerod (Chronicle et al. 2004; MacGregor et al. 2001; Ormerod et al. 2002) proposed a theory that describes how problems with ill-defined conditions and unclear goals are solved. In such problems, including classical insight problems, it is impossible to build a clear plan of action. However, progress is possible due to the formulation of local criteria for progress towards the goal and progress monitoring. Without having a clear goal and a clear plan of action, the solver still forms some expectations about the solution path and the outcome of the solution in the form of criteria for progress towards the goal and criteria for achieving the goal (a set of rather abstract expected properties of the goal state) (Chronicle et al. 2004).

We assume that the Aha! experience is a result of prediction errors regarding the criteria for achieving the goal. If the goal state, which eventually turns out to be the right solution, deviates from the initial predictions about the criteria for achieving the goal, an affective reward reaction occurs as pleasure and surprise being a signal to adjust predictions about the goal criteria for a more successful solution in the future. More accurate predictions about the criteria for achieving the goal state will allow the solver to build a more accurate solution plan and spend less time solving the problem.

Due to affective feedback of the Aha! experience, predictions are adjusted, and new knowledge is assimilated. Since the Aha! experience is usually the final point of the solution, the influence of the learned experience can manifest itself in other, similar problems. Therefore, the Aha! experience can be a necessary means of adjusting the criteria for achieving the goal in similar isomorphic problems.

However, how can one determine which problems and situations need to be adjusted and which do not? The solution structure can be transferred to similar problems (Day and Goldstone 2011; Gick and Holyoak 1983; Kurtz and Loewenstein 2007; Lee et al. 2015). Researchers in the field of transfer in problem-solving research demonstrated that solvers transfer their knowledge and solution methods based on the external features of similarity of the problems in conjunction with similarity of goals (Gick and Holyoak 1983; Zamani and Richard 2000). Therefore, we proceed from the fact that subjective expectations about the criteria for achieving the goal are also based on the external similarity and depend on past experience in solving similar problems. Thus, our main assumption is that in a set of similar isomorphic problems, solutions predictions become more accurate, which also entails a decrease in surprise and pleasure of the solutions.

## 2. Hypotheses

The current experiment was inspired by the studies of ‘mental set’ and insightful solution transfer (Luchins 1942; Öllinger et al. 2008; Bilalić et al. 2008). Within the mental set approach, an experimenter creates a set of isomorphic problems that consist of a sequence of problems with a similar solution principle. This set is usually followed by a critical problem which needs to be solved in a different, usually simpler way. The problem set decreases prediction error while increasing the expectation accuracy. The expectation accuracy is a solver’s subjective feeling of matching between initial and final criteria for achieving the goal. Specifically, each new problem becomes more and more expectable because it is solved in the same way, it uses the same criteria for achieving the goal, and it is built on the same principle as the previous one. 

We manipulated the expectedness variable by varying the similarity in problem solution principle and structure as follows: (1) different solution principle (DP), (2) same solution principle (SP), and (3) same structure and solution principle (SSP). The SP condition is designed to test whether expectations will be transferred to subsequent problems, despite the lack of external similarity between them. Expectations are built on the general solution principle, and the external similarity is simply an addition that allows the solver to quickly compare problems with each other and use the correct predictions. The SSP condition is designed to test whether successful application of expectations requires internal and external similarity of the problems. In this case, it is difficult to apply correct predictions without the external similarity of problems, hence the prediction error will not decrease. The DP condition represents a control.

There were eight different insight problems in the DP group. The SP and SSP groups had two parts of problems: (1) set part, consisting of seven problems. All problems in this part were similar to each other either by internal similarity or by external and internal similarities. (2) Critical problem (8th problem); it was different from problems of set part since it looked and was solved differently.

Our main hypotheses concern changes in expectation, pleasure, and surprise scales over the course of the problem sets. We assume that the greatest positive emotional reaction will be found in the situation of an unexpected solution that deviates slightly from the predicted one (for example, the 1st problem in all sets or the critical problem in the SP and SSP groups). The expected solution will not lead to strong positive reactions because it is fully consistent with the predictions and does not need the additional emotional reinforcement (for example, the 7th problem in the SP and SSP groups). In addition, we hypothesize that the expectedness of the solution relates to the learning that we can verify by estimating the solution time. More specific formulations of these hypotheses are presented below:

1. The expectation scale:
  - The 7th problem will be more expected than the 1st problem in the same principle (SP) and same structure and principle (SSP) groups but will not change in the different principle (DP) group.
  - The 8th problem will be more unexpected than the 7th problem in the same principle (SP) and same structure and principle (SSP) groups but not in the different principle (DP) group.
2. The pleasure scale:
  - The 7th problem will be less pleasurable than the 1st problem in the SP and SSP groups but not in the DP group.
  - The 8th problem will be more pleasurable than the 7th problem in the SP and SSP groups but not in the DP group.
3. The surprise scale:
  - The 7th problem will be less surprising than the 1st problem in the SP and SSP groups but not in the DP group.
  - The 8th problem will be more surprising than the 7th problem in the SP and SSP groups but not in the DP group.
4. The solution time:
  - The 7th problem will require less time than the 1st problem in the SP and SSP groups but will not change in the DP group.
  - The 8th problem will require more time than the 7th problem in the SP and SSP groups but not in the DP group, where the solution time of the 7th and 8th problems will be the same.

## 3. Method

In this experiment, we aimed to test the idea that the strength of the Aha! experience depends on the expectation accuracy. By the expectation accuracy, we mean subjective feeling of matching between initial and final criteria for achieving the goal.

### 3.1. Participants

Participants were: (1) in the DP group: 31 people (22 females), aged 18–56 years (*M* = 25.4, *SD* = 11.4); (2) in the SP group: 31 people (24 females), aged 18–42 years (*M* = 22.6, *SD* = 6.3); and (3) in the SSP group: 31 people (12 females), aged 17–52 years (*M* = 28.6, *SD* = 10.1). Most of the participants were undergraduate psychology students from the Yaroslavl State University. All participants were tested individually, took part voluntarily, and did not receive payments or credits for their participation. All participants gave written informed consent in accordance with the Declaration of Helsinki.

### 3.2. Stimuli

There were three experimental groups with different problem sets. The SP (same principle) group was presented with a set of eight insight problems: seven of them were isomorphic, i.e., the solution is carried out according to the same solution principle; the last (eighth) problem was critical. The solution of this set was based on the principle of analyzing letters. For example, the following problem was used: “Sally Lou likes eucalyptus more than pines. She likes electric lights and doesn’t like to sit by candlelight. Eccentric people cause her more sympathy than balanced ones. What do you think, who is Sally Lu by profession—an economist or an accountant?” The correct answer is an economist. The key to the answer is the analysis of letters: everything that Sally Lou likes begins with the letter e. The external structure of problems from this group were different from each other, hence participants could not guess that problems were solved in a similar way. The participant first needs to find the answer in order to make sure that its solution principle coincides with the previous problem. External structure does not tell the participant that the current problem is similar to the previous one. The last (eighth) problem was critical and had a unique solution principle. The following problem was used: “A man jumped out of a plane without a parachute. He landed on solid ground, but remained unharmed. Why?” The correct answer is the plane was on the ground. As we can see, the analysis of letters does not help in this way, because the solution principle is different.

In the SSP (same structure and principle) group, seven isomorphic problems with the same solution principle and problem structure were used. The solution principle was the analysis of letters, but, in addition, problems were similar in how their givens were formulated. For example, participants solved the first problem: “There is one in a minute and two in a moment, but only one in a million years. What is it?” (the letter m). Then, they received the second problem: “Every poet has it, but every artist exists without it, although an etude cannot be called an etude without it. What is it?” (the letter e). Each problem included the analysis of letters in three words and its answer was one of the letters. In this case, the problem structure helps to find similarity between problems before the answer has been found. The last (eighth) problem was the same critical problem as in the SP group.

The DP (different principles) group were presented with eight insight problems with various solution principles. There were problems with such difficulties as tautology, family relationships, attention to the wording, etc. For example, “Once two fathers and two sons found three oranges. They divided them among themselves so that each got one orange. How could this happen?” or “How can the number of 66 be increased by one and a half times without performing any arithmetic operations?”. The last (eighth) problem in this group was also critical and required the analysis of letters as in the SP and SSP groups: “The harp has four of them, the guitar and dombra have six, and the cello has five. What is it?”. The DP group is needed to verify that the emotional effects are associated with a change in expectations about the solution principle but not with the learning of insight problem solving. The full list of problems is presented in Table A1 of the Appendix A.

We also used a questionnaire to assess participants’ subjective feelings about the problem-solving process. The participants received six rating scales that they had to evaluate: pleasure, unpleasure, surprise, suddenness, certainty, and expectation. All rating scales were Likert scales with 7 points. As a basis, we took the Danek and Wiley’s questionnaire (2017), but with an additional scale about participants’ expectations: “I expected what the problem solution would be, I expected it (1)/I did not expect it (7)”. This scale was required to check whether participants were aware that the problems of SP and SSP groups were solved in a similar way. We also removed two scales (relief and drive) because, firstly, our hypotheses do not describe changes on these scales, and secondly, we did not want to increase the number of compared pairs in statistical processing. We divided the pleasure scale into two subscales: pleasure and unpleasure. In our opinion, the simultaneous presence of both positive and negative emotions is possible in the solution process of the same problem. These emotions are not rigidly linked and are not mutually exclusive. The scales were presented in a fixed order. We did not provide any theoretical frameworks or definitions to the participants. In addition, participants assessed their feelings in both cases when they found and did not find the solution. The final version of the questionnaire with all options can be found in OSF at https://osf.io/kpdfx. 

### 3.3. Procedure

The participants were tested individually. Each participant was randomly assigned to one of the groups: DP, SP, or SSP. Problems were printed on paper sheets. The participant’s goal was to solve the problems as quickly as possible. The time was limited by 5 min and was measured by a stopwatch. The participants solved the problems aloud without keeping any records (for example, on the paper). After finding the solution, the participant filled in their subjective ratings of the Aha! experience. After that, the participant moved on to the next problem. The procedure was repeated until all eight problems were solved. The sequence of problem presentation was randomized, except for the last (critical) problem, whose position was fixed in each group. If the participants did not find the solution of any problems in the group, they were told the answer. After that, they filled in a different version of the Aha! experience questionnaire for unsuccessful attempts. In this modified version, the questionnaire scales were modified as follows: instead of the phrase “At the moment of solution…”, it was written “At the moment I was told the answer…”, etc.

### 3.4. Design

We used a between-subject design with three independent groups (DP, SP, SSP) receiving different sets of insight problems. We also recorded which type of solution was demonstrated by our participants: endogenous (the solution was found by the participant) or induced solutions (the solution was reported by the experimenter). The solution time and the subjective ratings of the Aha! experience were used as dependent variables. The data that support the findings of this study are openly available in OSF at https://osf.io/kpdfx/ (accessed on 6 November 2022).

### 3.5. Data Processing

For data processing, we used ANOVA and independent sample *t*-test. The Holm–Bonferroni adjustment was used for multiple comparisons (we indicate the adjusted *p*-values). The correlation was made by the Spearman’s *rho* criteria.

## 4. Results

We aimed to test our assumptions about the relationship between the expectation accuracy and the strength of the Aha! experience, specifically, the feelings of surprise and pleasure. We conducted the experiment, varying the expectedness of the solution by the external (the problem structure) and internal (the solution principle) similarity of insight problems. We recorded the solution time and subjective ratings of various scales^1^.

### 4.1. Endogenous and Induced Insights

Most of the problems were successfully solved and the number of induced insights was not too large (see Table 1). The exception is the first problem in each group, where the number of endogenous and induced insights is almost the same. In this regard, we decided to compare them in the first problem. If there are no differences between solution types, then further analysis can be made without dividing problems by the solution type. If there are differences, then induced insights will be excluded from further analysis. Unfortunately, separate analysis for induced insights is not possible due to an insufficient number of cases (for example, there are only 2 cases of induced insight in the 8th problem of the SSP group).

Analysis of the first problem showed that there are significant differences between endogenous and induced insights in the next scales: pleasure, *F*(1, 84) = 41.21, *p* < .001, *η*^2^ = .315; unpleasure, *F*(1, 84) = 32.65, *p* < .001, *η*^2^ = .271; surprise, *F*(1, 84) = 7.95, *p* = .006, *η*^2^ = .085; certainty, *F*(1, 84) = 5.93, *p* = .017, *η*^2^ = .061; and expectation, *F*(1, 84) = 5.24, *p* = .025, *η*^2^ = .058. Based on these results, solutions with induced insight were excluded from further analyses.

### 4.2. Subjective Rating of Expectation

**Figure 1:**
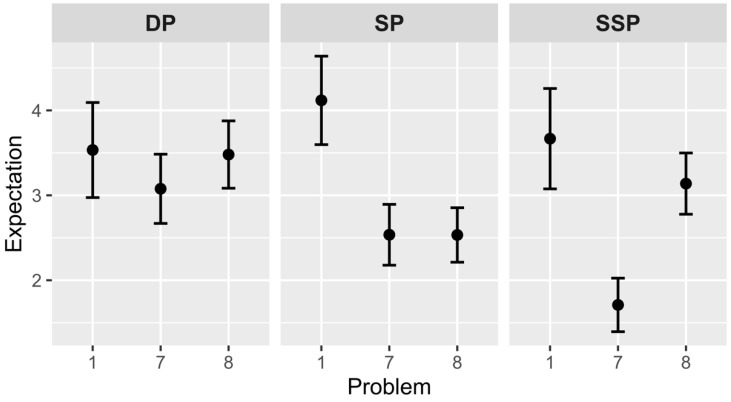
Subjective rating of expectations in different groups (1—expected solution, 7—unexpected). *Note.* Vertical bars denote standard errors. DP—different principle group, SP—same principle group, SSP—same structure and principle group.

Hypothesis 1 was tested using an independent *t*-test with the Holm–Bonferroni adjustment (see Figure 1). According to hypothesis 1a, the 7th problem in the SP and SSP groups will be more expected than the 1st problem. This hypothesis was confirmed following comparison of the 1st and 7th problems in the SP group (*M*_1_ = 4.1, *SD*_1_ = 2.1 and *M*_7_ = 2.5, *SD*_7_ = 1.9), *t*(43) = 2.58, *p* = .013, *Cohen’s d* = 0.794, and in the SSP group (*M*_1_ = 3.7, *SD*_1_ = 2.3 and *M*_7_ = 1.7, *SD*_7_ = 1.8), *t*(44) = 3.21, *p* = .003, *Cohen’s d* = 1.008. At the same time, expectations did not change in the DP group (*M*_1_ = 3.5, *SD*_1_ = 2.2 and *M*_7_ = 3.1, *SD*_7_ = 2.1), *t*(39) = 0.67, *p* = .509.

Hypothesis 1b that the 8th problem will be more unexpected than the 7th problem is correct only for the SSP group (*M*_7_ = 1.7, *SD*_7_ = 1.8 and *M*_8_ = 3.2, *SD*_8_ = 1.9), *t*(58) = –2.99, *p* = .004, *Cohen’s d* = –0.773. These differences were not significant for the DP group (*M*_7_ = 3.1, *SD*_7_ = 2.1 and *M*_8_ = 3.5, *SD*_8_ = 2), *t*(49) = –0.71, *p* = .482, and the SP group (*M*_7_ = 2.5, *SD*_7_ = 1.9 and *M*_8_ = 2.5, *SD*_8_ = 1.8), *t*(56) = 0.005, *p* = .996. In general, only the critical problem of the SSP group became more unexpected after the problem set.

Between-group analysis of the 7th problem showed that there is a significant influence of the *group* factor, *F*(2, 82) = 3.75, *p* = .028, *η*^2^ = .084. Post hoc comparisons with the Holm–Bonferroni adjustment found that the 7th problem of the SSP group is more expected than the 7th problem of the DP group, *p* = .025, *95% C.I.* = 0.2, 2.6. Analogous analysis of the 8th problem showed no significant differences.

### 4.3. Subjective Rating of Pleasure

**Figure 2:**
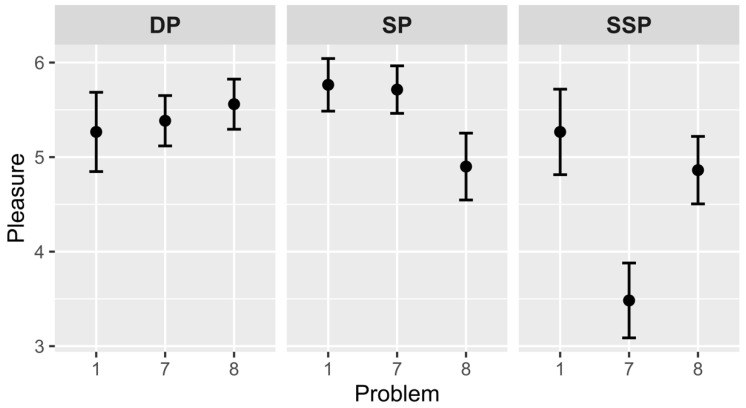
Subjective ratings of pleasure in different groups (1—unpleasure solution, 7—pleasure). *Note.* Vertical bars denote standard errors. DP—different principle group, SP—same principle group, SSP—same structure and principle group.

According to hypothesis 2a, the 7th problem will be less pleasurable than the 1st problem in the SP and SSP groups, but not in the DP group. The hypothesis was partially confirmed (see Figure 2). The differences between the 1st and 7th problems were not found for the DP group (*M*_1_ = 5.3, *SD*_1_ = 1.6 and *M*_7_ = 5.4, *SD*_7_ = 1.4), *t*(39) = –0.25, *p* = .804, and the SP group (*M*_1_ = 5.8, *SD*_1_ = 1.1 and *M*_7_ = 5.7, *SD*_7_ = 1.3), *t*(43) = 0.13, *p* = .897. The SSP group showed significant differences (*M*_1_ = 5.3, *SD*_1_ = 1.8 and *M*_7_ = 3.5, *SD*_7_ = 2.2), *t*(44) = 2.74, *p* = .009, *Cohen’s d* = 0.861.

Further, hypothesis 2b has been checked. This hypothesis suggests that the 8th (critical) problem will be assessed more pleasurable compared with the 7th problem in the SP and SSP groups, but not in the DP group. It was partially confirmed. The differences between the 7th and 8th problems were not significant for the DP group (*M*_7_ = 5.4, *SD*_7_ = 1.4 and *M*_8_ = 5.6, *SD*_8_ = 1.3), *t*(49) = –0.47, *p* = .643, and the SP group (*M*_7_ = 5.7, *SD*_7_ = 1.3 and *M*_8_ = 4.9, *SD*_8_ = 1.9), *t*(56) = 1.85, *p* = .069. However, the difference was found for the SSP group (*M*_7_ = 3.5, *SD*_7_ = 2.2 and *M*_8_ = 4.9, *SD*_8_ = 1.9), *t*(58) = –2.57, *p* = .013, *Cohen’s d* = –0.665.

Between-group analysis of the 7th problem showed that there is a significant influence of the *group* factor, *F*(2, 82) = 14.71, *p* < .001, *η*^2^ = .264. Post hoc comparisons with the Holm–Bonferroni adjustment found that the 7th problem of the SSP group is less pleasurable than the 7th problem of the DP group, *p* < .001, *95% C.I.* = 0.8, 2.9 and the 7th problem of the SP group, *p* < .001, *95% C.I.* = 1.2, 3.3. Analysis of the 8th problem showed no significant differences.

### 4.4. Subjective Rating of Surprise

**Figure 3:**
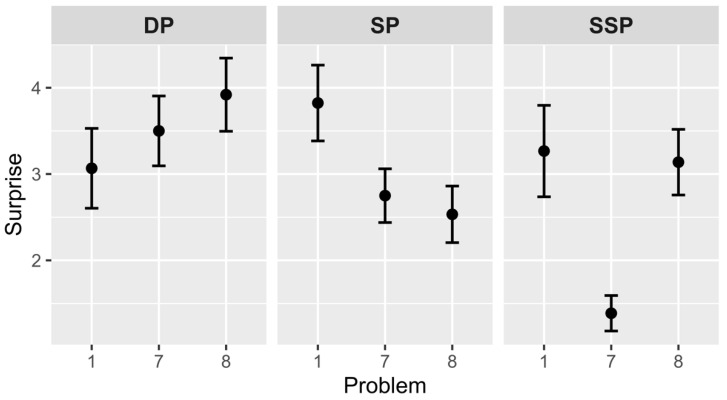
Subjective ratings of surprise in different groups (1—unsurprise solution, 7—surprise). *Note.* Vertical bars denote standard errors. DP—different principle group, SP—same principle group, SSP—same structure and principle group.

Hypothesis 3a suggests that the 7th problem will be less surprising than the 1st problem in the SP and SSP groups (see Figure 3). There were no differences in the DP group (*M*_1_ = 3.1, *SD*_1_ = 1.8 and *M*_7_ = 3.5, *SD*_7_ = 2.1), *t*(39) = –0.68, *p* = .502. The 1st and 7th problems were significantly different in the SP group (*M*_1_ = 3.8, *SD*_1_ = 1.8 and *M*_7_ = 2.8, *SD*_7_ = 1.6), *t*(43) = 2.04, *p* = .047, *Cohen’s d* = 0.628, and the SSP group (*M*_1_ = 3.3, *SD*_1_ = 2.1 and *M*_7_ = 1.4, *SD*_7_ = 1.1), *t*(44) = 4.00, *p* < .001, *Cohen’s d* = 1.258. Thus, the hypothesis was confirmed.

Hypothesis 3b is devoted to the 8th problem, which will be rated more surprising compared with the 7th problem in the SP and SSP groups. The differences between the 7th and 8th problems were not significant for the DP group (*M*_7_ = 3.5, *SD*_7_ = 2.1 and *M*_8_ = 3.9, *SD*_8_ = 2.1), *t*(49) = –0.72, *p* = .477, and the SP group (*M*_7_ = 2.8, *SD*_7_ = 1.6 and *M*_8_ = 2.5, *SD*_8_ = 1.8), *t*(56) = 0.48, *p* = .635. The 8th problem of the SSP group was more surprising than the 7th (*M*_7_ = 1.4, *SD*_7_ = 1.1 and *M*_8_ = 3.1, *SD*_8_ = 2), *t*(58) = –4.12, *p* < .001, *Cohen’s d* = –1.065.

Between-group analysis of the 7th problem showed that there is a significant influence of the *group* factor, *F*(2, 82) = 12.37, *p* < .001, *η*^2^ = .232. Post hoc comparisons with the Holm–Bonferroni adjustment found that the 7th problem of the SSP group is less surprising than the 7th problem of the DP group, *p* < .001, *95% C.I.* = 1.1, 3.2, and the 7th problem of the SP group, *p* = .004, *95% C.I.* = 0.3, 2.4. Analysis of the 8th problem also showed influence of the *group* factor, *F*(2, 81) = 3.33, *p* = .041, *η*^2^ = .076. The 8th problem of the DP group is assessed as more surprising than the 8th problem of the SP group, *p* = .035, *95% C.I.* = 0.1, 2.7.

### 4.5. Solution Time

**Figure 4:**
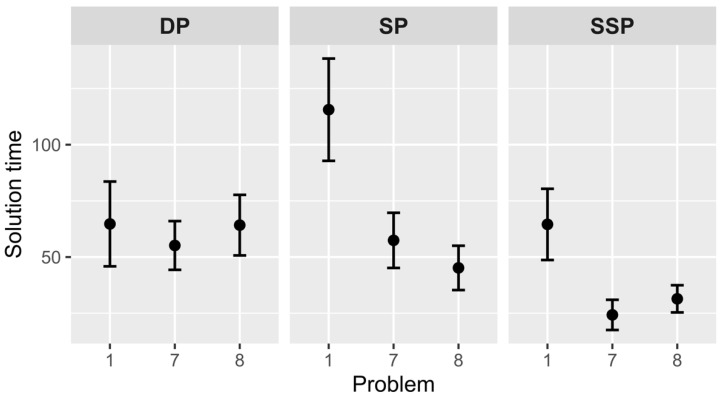
Solution times of different groups. *Note.* Vertical bars denote standard errors. DP—different principle group, SP—same principle group, SSP—same structure and principle group.

The solution time was analyzed only for successful solution attempts (see Figure 4). 

According to hypothesis 4a, the 7th problem will require less time than the 1st problem in the SP and SSP groups but will not change in the DP. It was entirely confirmed. The DP group did not show significant differences (*M*_1_ = 64.7, *SD*_1_ = 73.1 and *M*_7_ = 55.2, *SD*_7_ = 55.3), *t*(39) = 0.47, *p* = .638. However, there were differences in the SP (*M*_1_ = 115.6, *SD*_1_ = 93.8 and *M*_7_ = 57.4, *SD*_7_ = 65.0), *t*(43) = 2.46, *p* = .018, *Cohen’s d* = 0.755, and SSP groups (*M*_1_ = 64.5, *SD*_1_ = 61.4 and *M*_7_ = 24.3, *SD*_7_ = 37.4), *t*(44) = 2.76, *p* = .008, *Cohen’s d* = 0.868.

Hypothesis 4b suggests that the 8th problem will require more time than the 7th problem in the SP and SSP groups but not in the DP group. It was not confirmed for the SP, *t*(56) = 0.78, *p* = .437, and SSP groups, *t*(58) = –0.79, *p* = .434. Comparison of 7th and 8th problems of the DP group did not show significant differences as predicted, *t*(49) = –0.53, *p* = .602.

### 4.6. Correlation of Expectation, Pleasure and Surprise

The expectation, pleasure, and surprise were correlated to test their relationships. We used the Spearman’s *rho* criteria because the distribution differs from normal. Only data of successful attempts were used for this analysis. Significant positive correlation was found between the expectation and pleasure for all problems from 1st to 8th, *r*(605) = .134, *p* < .001, that is, the higher the pleasure of a problem, the greater unexpectedness is attributed to it and vice versa. Analysis of surprise revealed that the expectation also has a strong positive correlation with it, *r*(605) = .583, *p* < .001. 

## 5. Discussion

The main idea of this paper was that the strength of the Aha! experience varies depending on the expectedness of the solution. We made solutions more expected through sets of similar insight problems and hypothesized the decrease of their pleasure and surprise, because the unexpected solution would receive a stronger positive emotional reinforcement than the expected one.

### 5.1. Expectation

The hypotheses were consistently related to changes in the expectation, pleasure, and surprise scales of the Aha! experience, as well as the solution time. Subjective ratings of expectation predictably decrease, and solutions become more expected. The expectation decreases in problem sets with the same principle in both the SP and the SSP groups but not in the DP group. This result is fully consistent with our hypothesis. The strongest effect of expectation decrease is observed in the SSP groups: problems become more expected in the set part (the scale of expectation decreases), and the critical problem becomes more unexpected (the scale of expectation increases). This effect is less noticeable in the SP group, where the solution of the critical problem does not become more unexpected. It may be due to the fact that the critical problem subjectively looks the same as other problems of the set part.

### 5.2. Subjective Scales of Aha! Experience (Pleasure and Surprise)

We hypothesized that pleasure and surprise would decrease at the end of the set part in the SP and SSP groups because finding answers in these groups becomes more predictable and expectable due to external (problem structure) and internal (solution principle) similarities. The idea was confirmed: there was a decrease of surprise in the SP group, and both pleasure and surprise decreased in the SSP group. Consequently, the more expected the problem solution, the less it is supported by such feelings as pleasure and surprise. It is worth noting that pleasure decreased only in one group with both types of similarity (the SSP group). Probably—and it makes sense because these feelings are described in our languages in a very similar way—the subjective feeling of expectation is more strongly associated with surprise, hence changes in the surprise scale mirror changes in the expectation scale.

According to our hypotheses, the pleasure and surprise of critical problems increases after the set part of similar problems, but it happens only in the SSP group. The external similarity of problems from the SSP group influences the result because it helps the solver to separate familiar and similar problems from new ones which cause stronger affective reinforcement. The external similarity is an additional factor that allows the solver to make more accurate predictions.

### 5.3. Correlation of Expectation, Pleasure and Surprise

We assumed that there is a relationship between the expectedness of the solution, pleasure, and surprise. We found a significant positive correlation between the pleasure and the expectation for all problems. The more unexpected the solution, the stronger the pleasure. The surprise is also highly positively correlated with the expectation scale. Based on the correlation coefficient, it can be assumed that the key component of the relationship between the Aha! experience and the expectation is surprise. The surprise is a natural reaction to the discrepancy between expectations and results.

### 5.4. Solution Time

We assumed that the solution time of problems from SP and SSP groups would reduce, but the solution time of problems from the DP group would not change. The obtained data are in agreement with this hypothesis. We can observe the learning effect, i.e., the decrease of solution time from one problem to another: the greater similarity of problems in the set, the stronger the learning effect. The most surprising result is that the solution time of the critical problem does not increase after the set part of similar problems. Based on classical data (Luchins 1942; Öllinger et al. 2008; Bilalić et al. 2008), we expected to find an Einstellung effect in the SP and SSP groups. We believed it could manifest in the decrease of solution time in the set part and in the increase of solution time in the critical problem. However, critical problems also demonstrated low solution time. The obtained result probably indicates that participants are learning general problem-solving methods, rather than extracting specific knowledge about only one problem type. These general methods or heuristics equally effectively help in solutions for both similar and different problems. In this case, the heuristics may be the knowledge that all problems in the set have shortcuts; that it is necessary to look for a “second bottom” in the problem; and that it is necessary to read the problem conditions more carefully or not pay attention to numerical values because calculations do not help to find the answer. The result contradicts the data about the impossibility of spontaneous solution transfer between two problems (Barnett and Ceci 2002; Day and Goldstone 2011; Detterman 1993), because it shows that problems begin to be solved more and more efficiently in terms of solution time. The contradiction may be due to the fact that a typical transfer study is based on a comparison of two isomorphic problems, but in this case, a longer problem set was used. The presented work was not devoted to reasons and conditions that facilitate the solution transfer. 

## 6. General Discussion

Our model attempts to combine the subjective unexpectedness of insightful solutions, pleasure, and surprise as components of the Aha! experience and learning. Even solutions of ill-defined problems, part of which are insight problems, need a plan. It may include assumptions about what methods to use, what the result should be, and what are the criteria that a solution has been found. We assume that the solver starts problem solving with these assumptions from which they generate expectations acting as criteria for achieving the goal (Chronicle et al. 2004).

Using the approach of reward prediction error learning, we assumed that learning in the problem set occurs due to adjusting expectations. Expectations are adjusted by receiving emotional feedback in the form of the Aha! experience. The Aha! experience performs at least two functions in problem solving: (1) it is a subjective signal for adjusting expectations (surprise), and (2) it is one of the factors for remembering the useful experience gained in the form of emotional reinforcement (pleasure). The transfer of adjusted expectations is possible by catching the similarity between problems, which can be based on the solution principle and on the external features and problem structure. External features make it possible to evaluate the similarity of problems even before the solution starts, while it is possible to evaluate the internal similarity only after understanding the solution principle.

The obtained results confirmed this idea. The change in the expectedness entailed a change in the Aha! experience: the more expected the solution, the weaker the Aha! experience, and vice versa. The point is that predictions become more accurate and there is no need for emotional reinforcement. As we can see, the insight problem becomes less and less insightful if the expectedness of the solution increases. The problem loses positive emotional reinforcement and feeling of surprise after finding the answer. It does not happen if you solve various insight problems, as in the DP group. Thus, the similarity between problems is necessary to increase the degree of expectedness: the more features of similarity, the more expected will be the solution of the next problem.

The successful application of expectations requires the internal and the external similarity of problems. It is difficult for solvers to apply the correct predictions without external similarity (problem structure), which is why the pleasure as an affective component of prediction error is not reduced in the SP group. Insight problems in the SP group still have some novelty for the solver because their problem conditions and structure are very different from each other, requiring guessing their internal similarity. The solvers understand the similarity of the solution principle after they have successfully solved problems but not in the solution process. It can be a cause of the stronger Aha! experience. The external similarity of problem structure—as in the SSP group—helps to see the best start position for the solution. The problem structure is a kind of map, according to which we can easily understand how to build the best route.

Expectation also affected learning if we consider it as a decrease in the solution time. The problem set became increasingly expected, and at the same time, there was a decrease of the solution time. The solver could better predict promising moves, solve problems faster, and successfully learn to solve similar problems. This result was observed only in groups with external or internal problem similarity (the SP and SSP groups). Probably, the solver’s expectations are tied to specific features in the problem and cannot be generalized to a whole class of insight problems, as in the DP group.

Our results are consistent with findings of the pleasures of the mind approach, where the key feelings that accompany insight problem solving are the violation of expectation, curiosity, and virtuosity (Bianchi et al. 2022; Canestrari et al. 2018). Violation of expectation in problem solving is experienced as a tension caused by the unsuccessful attempts to solve a problem followed by the sense of relief caused by a solution; curiosity is experienced as a pleasure to learn something new; and virtuosity is a feeling of competence required to solve problems. The presence of these feelings in the solution of insight problems indicates that pleasure comes from unpredicted and unexpected solutions, which encourages the acquisition of new knowledge and increases self-confidence. Our findings are also in line with data obtained by Dubey et al. (2022) that suggested that metacognitive prediction errors can also lead to the Aha! experience.

Thus, we have shown that the strength of the Aha! experience is related to the degree of expectedness. The solution is evaluated as more insightful if it is unexpected; the solution is evaluated as less insightful if the solver expected the solution path. The Aha! experience is not an epiphenomenon. It performs its function in learning of problem solving through adjusting expectations.

## 7. Conclusions

In this paper, we aimed to test the relationship between the strength of the Aha! experience and the expectation accuracy. We found that these parameters are indeed related: the more unexpected the solution is, the stronger the Aha! experience. The expectation is built on the basis of two similarities—the external problem structure and the internal solution principle. The presence of only internal similarity cannot sufficiently reduce the pleasure of solution because there is a novelty that gives such feeling.

## Figures and Tables

**Table 1:** Percentage of endogenous solutions in different groups.

	Serial Number of Problem							
	1st	2nd	3rd	4th	5th	6th	7th	8th
Different principle	48.4	90.3	90	61.3	86.2	83.9	83.9	83.3
Same principle	60.7	80.7	90	76.7	77.4	87.1	90.3	96.8
Same structure and principle	48.4	77.4	83.9	93.5	93.5	100	100	93.5

## Data Availability

The data supporting results can be found in OSF at https://osf.io/kpdfx/.

## Note

1 This section presents the results of three subjective scales—expectation, pleasure and surprise. The results for the rest of the scales can be found at Table A2 of Appendix A.

## Appendix A

**Table A1:** Sets of problems in different experimental groups.

Different Principle	Same Principle	Same Structure and Principle
A father with a sly smile asks to his seven year old son: «Tell me the largest number». When he gets an answer, he just shakes his head in surprise, not knowing what to say. What number did the son say? *Solution: 31th, because the son thought about the number of days in the month (in Russian «number» is not only «digit», but also «date»)*	Sally Lou likes eucalyptus more than pines. She likes electric lights and doesn’t like to sit by candlelight. Eccentric people cause her more sympathy than balanced ones. What do you think, who is Sally Lu by profession—an economist or an accountant? *Solution: she is an economist. She prefers all with the first letter «E»*	It does not fit in the ocean, there is one in the sea, but there is two in the suitcase. What is it? *Solution: letter «S»*
According to statistics, 10% of all people prefer privacy and 80% of them have unlisted phone numbers. If you selected 400 names at random from the town’s phone directory, how many of these people selected would have unlisted phone numbers? *Solution: None, unlisted phone numbers are not in the directory*	It does not fit in the ocean, there is one in the sea, but there is two in the suitcase. What is it? *Solution: letter «S»*	There is one in a minute and two in a moment, but only one in a million years. What is it? *Solution: letter «M»*
Two people came to the river at the same time. However, the boat on which they can cross the river can support only one person. Each of them, without outside help, crossed on this boat to the other side. How did they do that? *Solution: two people were on opposite sides of the river*	Make one word from the set of letters below: R O W E D O N *Solution: one word*	There is the beginning of the end, the end of each segment, but does not occur in the middle. What is it? *Solution: It is Russian version of the problem: «I am the beginning of the end, and the end of time and space. I am essential to creation, and I surround every place. What am I?» The answer of Russian version is a letter «E» like in the English version*
The dog was tied to a 10 m rope but walked forward 300 m. How did she do it? *Solution: the rope was not tied*	Initially, this word consists of nine letters, but it can be written with two letters. Previously, it was written with ten letters, and now with three. What are we talking about? *Solution: it is «initially», «it», «previously» and «now» words*	The beginning of foundations, the middle of an aeon, but the end of space. What is it? *Solution: the problem is analog to the previous one. The answer of Russian version is letter «O»*
Once two fathers and two sons found three oranges. They divided them among themselves so that each got one orange. How could this happen? *Solution: there were three people (grandfather, father and son)*	Where does the wedlock go first, and only then the engagement? *Solution: dictionary*	Twice included in a circle, once in a triangle, but not included in a square. What is it? *Solution: the problem is analog to the previous one. The answer of Russian version is letter «O»*
How can the number of 66 be increased by one and a half times without performing any arithmetic operations? *Solution: it needs to be flipped*	There is a wrong word in the dictionary. What word is it? *Solution: wrong*	The girl and the man have it, but the woman is lost. What is it? *Solution: the problem is analog to the previous one. The answer of Russian version is letter «Ч [tch]»*
Three doctors have a brother Sergei, but Sergei has no brothers. How can this be? *Solution: doctors are women and Sergei’s sisters*	There is one in a minute and two in a moment, but only one in a million years. What is it? *Solution: letter «M»*	Every poet has it, but every artist exists without it, although an etude cannot be called an etude without it. What is it? *Solution: letter «E»*
The harp has four of them, the guitar and dombra have six, and the cello has five. What is it? (it is a critical problem) *Solution: letters*	A man jumped out of a plane without a parachute. He landed on solid ground, but remained unharmed. Why? (it is a critical problem) *Solution: the plane was on the ground*	

**Table A2:** Independent *t*-test of subjective scales.

Group	Subjective Scale	Serial Number of Problems	*M*	*SD*	*df*	*t*	*p-Level*	*Cohen’s d*
Different principle	Unpleasure	1 & 7	1.5	0.7	39	−0.40	.693	−0.13
			1.6	1.3				
		7 & 8	1.6	1.3	49	−0.37	.715	−0.10
			1.8	1.5				
	Suddenness	1 & 7	4.5	2.0	39	−0.68	.502	−0.22
			5.0	2.2				
		7 & 8	5.0	2.2	49	−0.43	.672	−0.12
			5.2	1.8				
	Certainty	1 & 7	5.8	1.7	39	−0.01	.989	−0.01
			5.8	1.7				
		7 & 8	5.8	1.7	49	−0.55	.584	−0.15
			6.0	1.3				
Same principle	Unpleasure	1 & 7	1.9	1.0	43	1.94	.059	0.60
			1.4	0.8				
		7 & 8	1.4	0.8	56	−0.94	.350	−0.25
			1.7	1.5				
	Suddenness	1 & 7	5.2	2.1	43	0.42	.679	0.13
			5.0	2.1				
		7 & 8	5.0	2.1	56	−1.64	.108	−0.43
			5.8	1.8				
	Certainty	1 & 7	5.4	1.7	43	−2.25	.030	−0.69
			6.4	1.1				
		7 & 8	6.4	1.1	56	1.01	.317	0.27
			6.0	1.3				
Same structure & principle	Unpleasure	1 & 7	1.3	0.8	44	−1.69	.099	−0.53
			2.2	2.0				
		7 & 8	2.2	2.0	58	1.97	.054	0.51
			1.4	0.9				
	Suddenness	1 & 7	5.2	1.9	44	−0.44	.664	−0.14
			5.5	2.5				
		7 & 8	5.5	2.5	58	0.29	.777	0.07
			5.3	2.2				
	Certainty	1 & 7	5.9	1.1	44	−1.49	.143	−0.47
			6.5	1.2				
		7 & 8	6.5	1.2	58	1.31	.196	0.34
			6.0	1.5				
